# Personal

**Published:** 1909-02

**Authors:** 


### PERSONAL.

Dr. Charles G. Stockton, of Buffalo, was elected president of the Medical Society of the State of New York, at its one hundred and third annual meeting held at Albany, January 25-27, 1909. Dr. Stockton is in the prime of active professional life, is Professor of Medicine at the University of Buffalo, and is the most conspicuous representative of what is called nowadays internal medicine, who has been chosen in recent years to this high office.

Dr. Stockton is a teacher of renown, popular with his classes, and thorough in his methods. He is imbued with the science of medicine, and imparts it with forceful epigrams, or extended sentences, as may seem best, but always in graceful English that conveys a lasting impression. He is also an author who employs a facile pen, that gives to a larger audience the benefit of his great experience in the study of disease and its most skilful method of treatment. The society with its 6,000 members as well as the profession at large is to be congratulated on the selection of such a leader.

---

THE President closed a conference Saturday, January 23, 1909, with Dr. Albert Ferris, president of the New York State Commission in Lunacy, and Dr. Sydney D. Wilgus, chief examiner of the state board of alienists, relative to the imposition of heavier penalties for bringing insane aliens into the country.

---

DR. WILLIAM C. KRAUSS, of Buffalo, who has been in California for some weeks will return February 1, and take up his professional work.

---

DR. AND MRS. F. PARK LEWIS, of Buffalo, will sail late in February for a recreation tour in Europe, returning in May.

---

DR. ERNEST WENDE, Health Commissioner of Buffalo, and professor of dermatology at the University of Buffalo, is at the Buffalo General Hospital suffering from severe illness. We trust his recovery is to be anticipated at an early day.

